# A Case of Treatment-Resistant Schizophrenia With Mesial Temporal Sclerosis: A Case Report

**DOI:** 10.7759/cureus.49623

**Published:** 2023-11-29

**Authors:** Shokry Alemam, Syed Ali Bokhari, Safa F Hasan, Sara Al Ammour, Basma Hussein, Muhanad Elnoor

**Affiliations:** 1 Psychiatry, Al Amal Psychiatric Hospital, Dubai, ARE

**Keywords:** refractory complex partial seizures, schizophrenia and temporal lobe, mesial temporal lobe epilepsy, mesial temporal sclerosis (mts), treatment resistant schizophrenia

## Abstract

Mesial temporal sclerosis (MTS) is one of the most common causes of treatment-resistant epilepsy, especially temporal lobe epilepsy (TLE). Various psychiatric symptoms are common with temporal lobe epilepsy. However, the least established symptoms were psychotic symptoms. Furthermore, treatment-resistant schizophrenia is a significant proportion of schizophrenia patients who have failed treatment with at least two different antipsychotics, resulting in poor outcomes and a significant negative impact on the patient's life.

In our case report, psychotic symptoms and abnormal behaviors were explained by schizophrenia for more than 17 years in a 32-year-old female, while the diagnosis of temporal lobe epilepsy with mesial temporal sclerosis was missed, resulting in incomplete treatment, which led to a deterioration of her quality of life for years.

This case aims to shed light on TLE rare manifestations and to discuss the proper investigations and treatment that might increase the quality of life of these patients. Underlining the necessity for more research in treatment-resistant schizophrenia, this unusual case underscores the importance of exploring the underlying biological, psychological, and social risk factors. It also emphasizes the need to focus additional attention on formulating proper investigation strategies for the susceptible patient population.

## Introduction

Mesial temporal sclerosis is the scarring and loss of neurons and nerve cells in the deep part of the temporal lobe with hippocampal sclerosis and is commonly associated with seizures. 60% to 80% of drug-resistant temporal lobe epilepsy (TLE) cases are associated with mesial temporal sclerosis (MTS); therefore, it is considered the most recognized pathology [1].

The limbic system, which is involved in TLE, significantly regulates mood, emotions, and behavior. Therefore, TLE patients have a high chance of presenting with psychiatric disorders [2]. However, the prevalence of previously reported cases of TLE with psychotic and behavioral symptoms is less than 1% [3]. Literature shows a higher incidence of psychiatric disorders among patients with TLE in comparison with generalized epilepsy (79% vs. 49%), and in a more recent study, it was highlighted that psychosis has a higher incidence [4,5]. The predisposing factors of psychosis with mesial temporal lobe epilepsy (MTLE) are not fully understood; nevertheless, postictal psychosis (PIP), in 7% to 10% of TLE cases, has been associated with several factors, including longer duration of epilepsy, positive family history of psychosis, impaired intellectual function, and gross structural lesions [6-8]. MTS etiology is not fully understood; however, Wyler et al. [9] found Pyramidal cell loss in hippocampal subfields CA1-CA4. 

The gold standard investigation for diagnosing MTS is recognized to be magnetic resonance imaging (MRI) [10]. The success rate of medical treatment is only 25%, while the surgical intervention can be considered more effective with 70-95% of the patients undergoing tailored anterior temporal lobectomy, including amygdala-hippocampectomy with the majority of the cases being seizure-free in the first 2 years after surgery [11].

This case aims to shed light on TLE manifestations and to discuss the proper investigations and treatment that might improve the quality of life of these patients.

## Case presentation

A 32-year-old female patient, who is single and has never been employed previously, presented with her family complaining of a 17-year history of hearing voices and seeing unknown people while she is alone in her room, in addition to smelling bad odors in her room and her clothes on multiple occasions. Also, she believed that others monitor and talk about her. Moreover, she complained of difficulty falling asleep and fragmented sleep. She had been socially withdrawn and had poor hygiene. 

Her history revealed sudden agitation with verbal and physical aggression with no specific precipitating or alleviating factors. Also, she had bouts of diurnal enuresis along with disorientation to time and place. Also, bouts of disinhibition, such as running out of the house in sleeping clothes, were reported. She did not have any past medical history. About the family history, her maternal cousin had similar symptoms, such as visual hallucinations, olfactory hallucinations, abnormal behavior, and bouts of aggression. However, he was never formally diagnosed.

The patient sought medical advice for the first time when she was 15 years old, and she received different diagnoses, including bipolar affective disorder and schizoaffective disorder, based on the bouts of disinhibition and agitation. Moreover, she had been diagnosed with schizophrenia, substantiated by the visual and auditory hallucinations as well as delusions of reference and persecution against her family members and housekeepers. Over the years of her treatment, she was commenced on multiple medications, including Risperidone 3 mg tablets, Trifluoperazine 10 mg, Fluoxetine 20 mg, Aripiprazole 30 mg, Olanzapine 15 mg, Risperidone long-acting injection 50 mg every two weeks, Lamotrigine 100 mg, and Haloperidol 20 mg.

On assessment, the patient was overweight and had a bizarre appearance. She had poor attention and concentration, which needed redirection during the assessment. She had coherent speech of decreased volume and pressure. She described her mood as fair despite having blunted affect. Her thinking process was concrete with the poverty of thoughts. Her thought content included delusions of reference and persecution. She reported visual, auditory, and olfactory hallucinations. She had no insight or judgment. The patient denied death wishes, suicidal thoughts, plans, or intent.

During later visits, she reported recurrent abdominal pain, which preceded her bouts of agitation and aggression, which she couldn't recall but was informed by her family, for which abdominal ultrasound, CT, and other routine blood investigations were done, revealing normal results. Her family reported repetition of these episodes multiple times daily.

**Figure 1:**
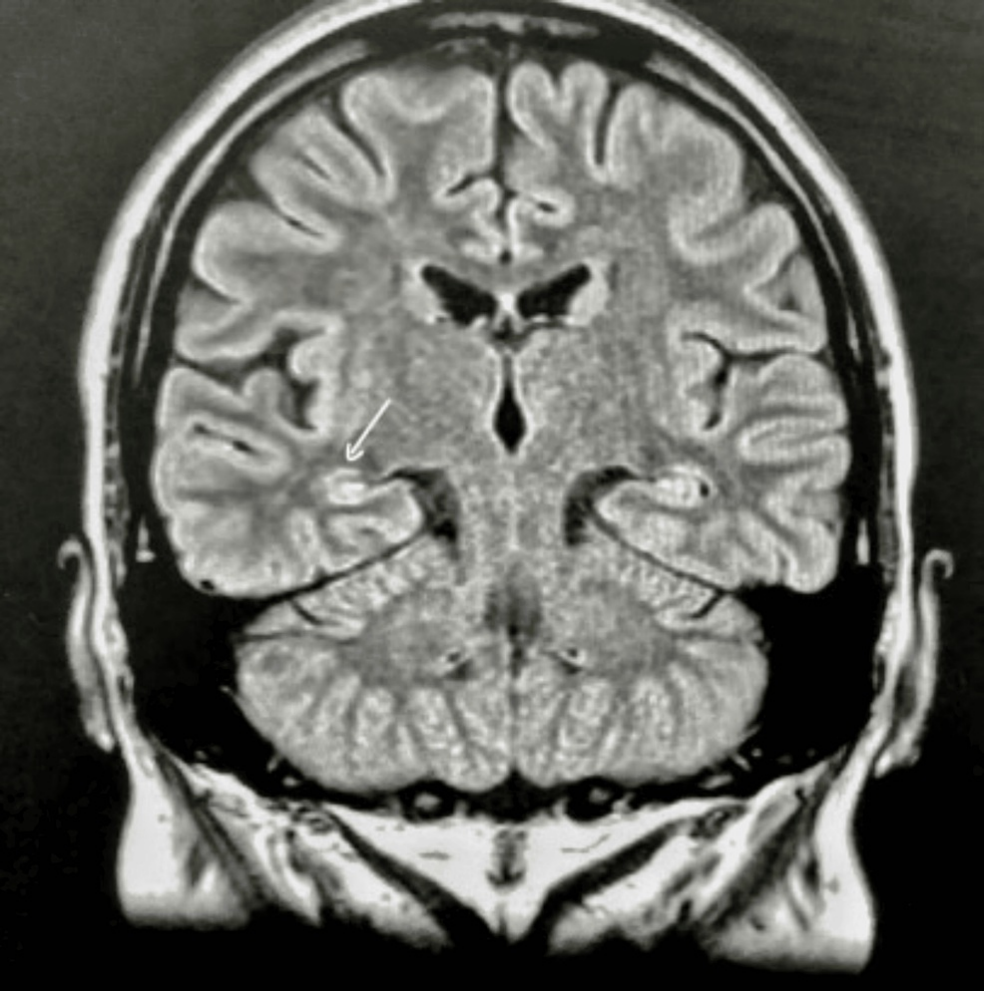
Shows a decrease in the size of the right hippocampus compared to the left side.

A brain MRI showed venous malformation at the right side of the cerebellum and decreased volume of the right hippocampus and right mesial temporal sclerosis (Figure 1). EEG was unremarkable; the patient was irritable and did not agree to do a video EEG.

A diagnosis of complex partial seizure with mesial temporal sclerosis was made in addition to the treatment-resistant schizophrenia. Therefore, all the medications were discontinued gradually over two weeks, and she was started on clonazepam with gradual titration of up to 3 mg daily to control her irritability and use the anti-epileptic effect of clonazepam. Two weeks later, Ms. S showed partial improvement in concentration, orientation to place and person, irritability, agitation, abdominal pain, and olfactory hallucinations. However, delusions and auditory hallucinations remained with no improvement. Clonazepam was continued along with the initiation of Clozapine up to 300 mg. At a later stage, oxcarbazepine was added and gradually titrated up to 1200 mg. 

Currently, the patient has a good response regarding the complex partial seizures as the frequency decreased from multiple times a day to once or twice a month; however, the delusions and hallucinations remain with less intensity and frequency, which do not interfere with the patient's daily life activities. 

## Discussion

The association between epilepsy and psychiatric disorders has been under the scope of study. Many articles have been published looking at their prevalence and the possible pathophysiology linking the two conditions. In pharmaco-resistant epilepsy patients assessed for epilepsy surgery, there was an occurrence of 22% of affective disorders [12]. In (mostly pharmaco-resistant) focal epilepsy, the prevalence of a lifetime history of depression was much higher, at 40% [13]. In patients with pharmaco-resistant TLE-MTS, the prevalence of major depressive disorder was 22.1%, significantly higher than in healthy individuals from the community [14]. This was not the case with our patient, as the prominent symptoms in our case were psychotic symptoms. She did not have any mood disorder, which makes this case quite significant.

Epileptic patients are more likely to experience psychosis, establishing a reciprocal relationship where epilepsy becomes a risk factor for psychosis and vice versa [15,16]. There was a prevalence of 4% of psychotic disorders (schizophrenia, schizoaffective disorder, other psychosis) in pharmaco-resistant epilepsy [12]. Clancy et al.'s 2014 systematic review revealed that the combined prevalence of psychosis in individuals with epilepsy was 5.2%. This figure increased by 13% in complex partial seizures and ranged from 7% to 12% higher with temporal lobe epilepsy [15,17-20]. Temporal lobe epilepsy (TLE) has a probability of more than 70% to be associated with abdominal aura [21], which was seen in our case.

Mesial temporal sclerosis serves as the predominant pathological condition linked to intractable temporal lobe epilepsy (TLE), observed in approximately 60-80% of cases [1].

It is crucial to keep in mind that psychiatric symptoms can occur during the different phases of seizures. The prevalence of postictal psychosis has been estimated to be 6-10% in patients specifically with temporal lobe epilepsy [22]. The symptoms could include delusions, hallucinations, and even thought abnormalities, which are usually transient but can last several weeks. Those symptoms, which could mimic functional mental illness, can make diagnosis difficult. Risk factors that increase susceptibility to psychotic symptoms in the postictal stage include bilateral epileptic foci, ictal fear, or gross structural lesions. Managing acute postictal psychosis may necessitate the need for short-term courses of antipsychotics or benzodiazepines. Improving seizure control would be the long-term goal [23].

In addition to postictal psychosis, the prevalence of inter-ictal psychosis in patients with epilepsy, particularly those with temporal lobe epilepsy, has been documented to range from 4-10% [24]. Moreover, the occurrence of inter-ictal psychosis in pharmaco-resistant TLE-MTS was 5% [14]. Reported risk factors include bilateral temporal foci, the early onset of epilepsy, and a persistent refractory course. Unlike postictal psychosis, inter-ictal psychosis requires treatment with antipsychotic medications, preferably second-generation, which was the case with our patient after receiving the diagnosis of MTS-TLE, leading to a better outcome of her final treatment. Psychosocial support and family education are also essential to support the patient.

Clozapine treatment is recommended for an indefinite period as the patient was being aggressive and had frequent relapses. Clozapine had the same level of evidence as Olanzapine and Quetiapine, which are recommended for the treatment of psychotic-like symptoms related to epilepsy [25].

Although our patient used to have seizures multiple times a day, risk factors for poor seizure control, such as early age of seizure onset, a history of febrile convulsions, and epileptiform discharges on the EEG [26], were not present in our case. Proper investigation and review of the history, as well as treatment plan, revealed the diagnosis of hippocampal atrophy and MTS, which was a significant factor for her TLE. It is well-known that Clozapine can lower the seizure threshold. The risk of seizures with Clozapine doses of 300 mg to 600 mg is 2.7% [27]. However, the patient achieved significant improvement in her psychotic symptoms and a reduction in seizures to only once or twice per month.

## Conclusions

In conclusion, treatment-resistant psychosis and mesial temporal sclerosis can both deteriorate a patient's quality of life, as mesial temporal sclerosis is resistant to pharmacological treatment. Thus, a thorough history and appropriate investigations of treatment-resistant psychosis can lead to a better understanding of the cause and comorbidities, leading to a better management plan and thus improving the patient's quality of life. This case report warrants an increase in awareness about the need to investigate any sudden onset of psychosis to rule out organicity. Moreover, further research is needed to understand the connection between MTS-TLE and chronic psychosis to improve the patient's quality of life.
